# Comparison of Lateral Abdominal Musculature Activation during Expiration with an Expiratory Flow Control Device Versus the Abdominal Drawing-in Maneuver in Healthy Women: A Cross-Sectional Observational Pilot Study

**DOI:** 10.3390/medicina56020084

**Published:** 2020-02-19

**Authors:** Vanesa Abuín-Porras, Paula Maldonado-Tello, Mónica de la Cueva-Reguera, David Rodríguez-Sanz, César Calvo-Lobo, Daniel López-López, Emmanuel Navarro-Flores, Carlos Romero-Morales

**Affiliations:** 1 Faculty of Sport Sciences, Universidad Europea de Madrid, Villaviciosa de Odón, 28670 Madrid, Spainpaula_865@hotmail.com (P.M.-T.); monica.delacueva@universidadeuropea.es (M.d.l.C.-R.); 2 Facultad de Enfermería, Fisioterapia y Podología, Universidad Complutense de Madrid, 28040 Madrid, Spain; davidrodriguezsanz@gmail.com (D.R.-S.); cescalvo@ucm.es (C.C.-L.); 3 Research, Health and Podiatry Group, Department of Health Sciences, Faculty of Nursing and Podiatry, Universidade da Coruña. La Coruña, 15403 Ferrol, Spain; daniellopez@udc.es; 4 Department of Nursing, Faculty of Nursing and Podiatry, Frailty and Cognitive Impairment Organized Group (FROG). University of Valencia, 46001 Valencia, Spain

**Keywords:** ultrasonography, abdominal muscles, abdominal drawing-in maneuver, muscle activity

## Abstract

*Background and Objectives:* The purpose of the present study was to quantify and compare lateral abdominal musculature thickness, including the transverse abdominis (TrA), internal oblique (IO), and external oblique (EO) muscles, via rehabilitative ultrasound imaging (RUSI) during the use of the expiratory flow control device (EFCD) versus the classic abdominal drawing-in maneuver (ADIM). *Materials and Methods:* A cross-sectional observational pilot study. Twenty-one women were recruited and assessed the thickness of each muscle (TrA, IO, and EO) by ultrasound imaging at rest, during the ADIM, and during expiration with the EFCD. Waist circumference was also measured under the same circumstances. *Results:* Statistically significant differences were observed between ADIM, EFCD, and at rest condition for the thickness of the TrA (*p* = 0.001) and IO (*p* = 0.039). Moreover, statistically significant differences for TrAb at rest compared with the ADIM (*p* = 0.001, Cohen’s *d* = 2.183) and at rest and with the EFCD (*p* = 0.001, Cohen’s *d* = 2.843). In addition, between ADIM and EFCD were not statistically significant, although a moderate effect size was found (*p* = 0.055, Cohen’s *d* = 0.694). For the IO muscle thickness, significant differences were reported between the EFCD and at rest (*p* = 0.038), Cohen’s *d* = 0.081). *Conclusions:* Significant differences in the increase of the thickness of the TrA and IO muscles during the use of the EFCD and the ADIM with respect to rest. In addition, for the TrA, statistically significant differences were found during expiration with the EFCD with respect to the ADIM. Expiration with EFCD can be a useful method for the activation of the TrA.

## 1. Introduction

The lateral musculature of the abdomen comprises, from deep to superficial, the transverse abdominis (TrA), internal oblique (IO), and external oblique (EO) muscles [1].

The TrA is a part of the deep stabilization system of the trunk, along with the multifidus, the diaphragm, and the pelvic floor musculature [2,3,4]. These muscle activations cause an increase of the intra-abdominal pressure, contributing to lumbopelvic stabilization [5]. In patients with lower back pain, there is a change in abdominal muscle recruitment during activities demanding trunk stability [6,7,8]. The TrA can also be considered the most powerful musculature during expiration by increasing abdominal pressure and pushing the diaphragm towards the thorax, especially in forced expiration [6]. There is also a close relationship between the musculature of the pelvic floor and the abdominal musculature [7]. For example, an activation of the core musculature generates an increase in the bladder neck muscle (continence mechanism) [8]. Therefore, the TrA, within the lateral abdominal musculature, presents a predominant role in both lumbopelvic stabilization [3] and expiration [6], as well as during the synergy with the pelvic floor [8].

**Figure 1:**
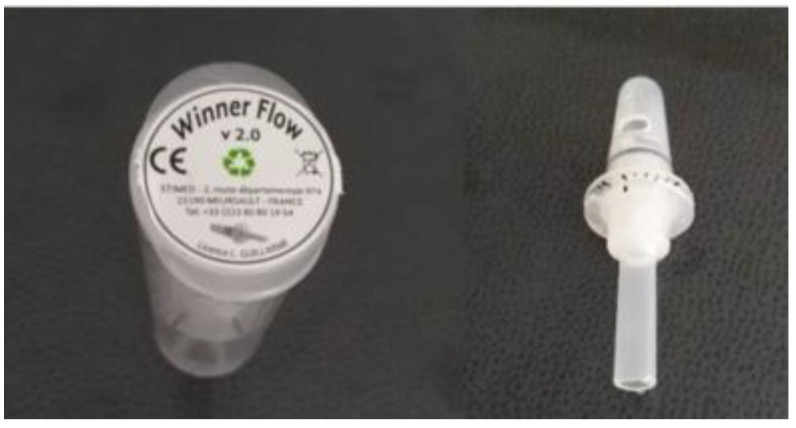
Expiratory Flow Control Device (EFCD) (Winner Flow, v2.0).

The abdominal drawing-in maneuver (ADIM) is frequently used to facilitate the activation of the TrA [3,4], employing the lower part of the abdominal wall, drawing the belly button towards the back [9], being effective in lumbopelvic stabilization programs [10], strengthening programs [11,12], improving pulmonary function [13], and in the reduction of lumbar pain symptoms [14]. Due to the difficulty of the ADIM execution, several authors have explored the TrA muscle activation using the expiratory muscle recruitment and finding benefits with respect to the execution of an ADIM, in the increase of the thickness of the TrA when performing maximal expiration [13,14,15]. To guide this expiratory flow, an exsufflation nozzle was developed and tested in urinary incontinence [16], an instrument for performing active expiration through an 8-mm tube, which maintains the glottis open. The expiratory flow control device (EFCD)—Winner Flow^®^ version. 2.0 (Prim Fisioterapia y Rehabilitación, Madrid, Spain) (Figure 1)—is an evolution of this first model [16], and was developed to redirect pressure, and based on abdominal-perineal synergy and the expiratory function of the TrA, an adjustable increase in expiratory flow may be generated [17].

Rehabilitative ultrasound imaging (RUSI), commonly used for the study of muscle morphology and characteristics, has been shown as a technique which presents good reliability with regard to trunk stabilizers [18]. The validity of ultrasound measurements of this musculature has been compared with measurements using magnetic resonance imaging (MRI), obtaining an intercorrelation coefficient (ICC) between 0.78 and 0.95 [3]. Moreover, RUSI can detect changes in TrA and IO activity related to changes in their thickness [19]. In addition, the use of ultrasound to assess the changes produced in the TrA, IO, and EO during an ADIM [3,19,20] and during maximal expiration [9,21] has been validated.

In this study, RUSI was used to compare lateral abdominal musculature thickness using ADIM versus a forced expiration through the EFCD with the purpose of evaluate the effects on the core muscles of a directed and regulated expiration by an EFCD device with respect to the ADIM.

## 2. Materials and Methods

### 2.1. Design

A pilot study was developed with a cross-sectional observational design following the Strengthening the Reporting of Observational Studies in Epidemiology (STROBE) guidelines. The study was conducted at the European University of Madrid (Madrid).

### 2.2. Ethical and Legal Considerations

The study was approved by the Intervention Clinical Committee of the European University of Madrid, Spain (CIPI/19/003; 10 February 2019). The present study adhered to the ethical standards of the Declaration of Helsinki. In addition, the consent inform form was obtained from all subjects before beginning this study.

### 2.3. Subjects

**Figure 2:**
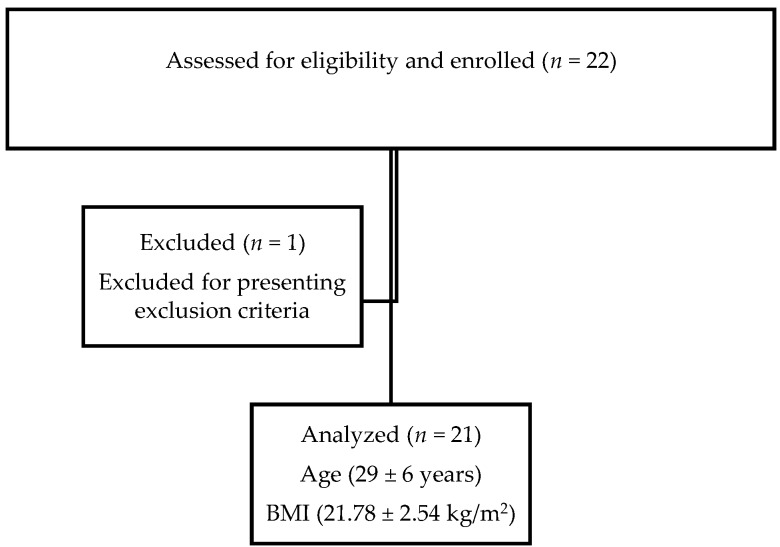
STROBE flowchart for recruitment. Body mass index, BMI.

A total sample of 21 female volunteers were recruited through an advertising campaign promoted by the European University Research Lab in order to recruit students and teachers of the University, from February to June (2019) (Figure 2). All participants signed the consent form after providing them all information in an appropriate environment to solve any doubts regarding this research. The inclusion criteria were: adult women (>18) years [22] and students and/or workers of the European University of Madrid at the time of the study. The exclusion criteria were: current lumbopelvic pain or history of lumbar pain in the 6 months prior to the study [23], body mass index greater than 31 kg/m^2^, respiratory disease, radiculopathy or neurological symptoms, neuromuscular diseases diagnosed [24], surgery of the lumbar or abdominal spine, severe instability or structural impairment that affects the lumbopelvic region [25], osteoporosis, systemic inflammatory diseases, severe cardiovascular pathologies, pathologies that affect the lower extremities such as fractures, surgeries or neoplastic disorders [24], inability to follow instructions, pregnancy [22,24], and high intensity physical activity or competition for more than three times a week [21,24].

The EFCD device and every tool needed in the study were provided by the European University of Madrid and its Research Lab department.

### 2.4. Outcome Measurements

The measurement of the dependent variables in this study was performed using an ultrasound device for the thickness of the lateral abdominal musculature and a measuring tape for the waist circumference. All measurements were performed by the same therapist, with two years of experience in ultrasound imaging. An ultrasound tool (LOGIC S7, XDclear, GE Healthcare, Little Chalfont, UK) and a linear transducer with a frequency range of 5 to 13 MHz (ML6-15, 38 mm transducer) were used during this study to obtain images in B-mode. A SECA brand metric tape, (model 201; CE 0123; SECA Deuchland, Hamburg, Germany) was used to measure waist circumference. Measurement of waist circumference was based on the fact that the lateral musculature of the abdomen, when activated, would form a “belt” or corset, producing a decrease in the horizontal diameter of the waist [25].

For the ADIM, with the subjects laying in a supine position and a hip flexion of 45° [4], the following instructions were given: “move your bellybutton inwards towards your back and hold it for 10 s. Do not move your pelvis nor your back” [14]. In addition, it was indicated that throughout the exercise, effort should be smooth according to the Borg scale, that is, that the effort perceived by the participant should be 2 on a scale of 0 to 10, with 0 being rest and 10 being the maximum effort [4].

The EFCD was placed between the lips. Following its protocol of use, the caliber was adjusted to each participant. To do this, as before, the participant was asked to exhale through the mouthpiece in supine decubitus and with 45° hip flexion without drawing air deeply beforehand. Participants were instructed to exhale normally during the 10 s that the EFCD was used. The first caliber to be used is the one labeled as “1”. If the expiration time did not reach the stipulated 4–5 s, the caliber was reduced one point at a time until this expiration time was reached. If, on the contrary, the stipulated 5 s were easily achieved, the caliber was increased. The expiration must be regular over 10 s to consider the caliber as adequate, with special observation to the abdomen. Total isometric contraction of the abdominal wall (bracing) was not allowed [17].

Ultrasound measurements were taken following the Whittaker et al. [24] guidelines, with the subjects in supine position with 45° hip and knee flexion [4]. A linear probe (ML6-15) was placed on the right side of the abdomen in a transverse plane, taking as reference the mid-axillary line and the midpoint between the iliac crest and the bottom of the rib cage for the muscle thickness measurements, the thickness of each muscle (TrA, IO, and EO) was measured between the inner edges of the fascial tissue [24]. Thus, the following images were taken to subsequently perform ultrasound measurements [24]: (1) Firstly, three images at rest, taken at the end of normal expiration. The thickness of the TrA, IO, and EO were measured in each image, and the mean of the three measurements for each muscle were calculated for further analysis [24]. (2) Secondly, three images were taken during three ADIMs, which were executed for 10 s, with each image being captured in the 10th second [26]—The thickness of the TrA, IO, and EO in each of the images was measured, and the mean for each muscle was calculated for further analysis [24]. (3) Finally, three images were taken for 3 expirations with the EFCD, which were executed for 10 s, with each image captured in the 10th second [26]—The thickness of the TrA, IO, and EO was measured in each image, and the mean for each muscle and the percent of change between rest and activation was calculated for further analysis following the protocol developed in the study by Ishida et al. [21,24].

To calculate the percent change in thickness of each of the 3 muscles during the ADIM or during expiration with EFCD with respect to thickness during rest, the following formula was used, developed in the study by Ishida et al. [21]: (ADIM or EFCD Thickness—Thickness during rest/Thickness during rest × 100) (%). The average thickness was obtained for each muscle during the ADIM, during the use of the EFCD and at rest [21]. Likewise, to calculate the percent change in waist circumference during the ADIM or expiration with the EFCD with respect to waist circumference during rest, the following formula was used [21], using the mean waist circumference during the ADIM, during the use of the EFCD and during rest: (ADIM or EFCD Circumference—Circumference during rest/Circumference during rest × 100) (%).

Waist circumference was measured with the participant in supine decubitus with a 45° hip flexion; the measuring tape was placed at the level of the bellybutton, taking as reference the midpoint between the last rib and the upper edge of the iliac crest [27], passing it behind the back and around the waist [28]. The following measurements were obtained: (1) Three measurements at rest, at the end of normal expiration [27]; (2) Three measurements at the end of each ADIM, with each maneuver lasting 10 s; (3) Three measurements at the end of expiration through the EFCD, with each expiration lasting 10 s. The mean of each of the 3 measurements in the different situations was later analyzed, and the percent of change between rest and activation was calculated.

The participants were examined in one single session in the same day. The examination time was 30 min approximately. The order of the execution of the ADIM and the EFCD was randomized, with 10 min between the two tests. 

### 2.5. Statistical Analysis 

The data resulting from the study were analyzed using the statistical program SPSS (SPSS Version 23.0, IBM, Armonk, NY, USA). Throughout the study, we used an α error of 0.05, a β error of 0.02, and a confidence interval of 95%. First, the Shapiro–Wilk test was performed to assess the normality. Second, the descriptive analysis for the total sample was carried out. At last, a comparative analysis between EFCD, ADIM, and at rest was performed by a One-Way Analysis of Variance (ANOVA). In addition, the effect size was estimated with the Cohen’s *d* formula: (M2 − M1)/standard deviation (SD). For the effect size, Cohen suggested that d = 0.2 be considered a ‘small’ effect size, 0.5 represents a ‘medium’ effect size, and 0.8 a ‘large’ effect size.

## 3. Results

**Figure 3:**
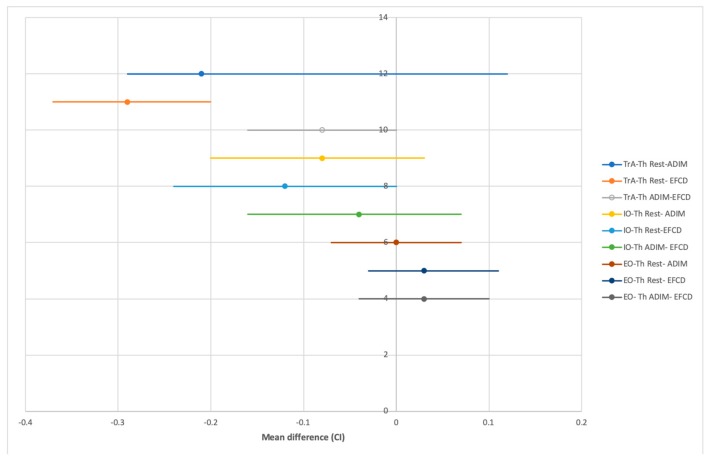
Abdominal drawing-in maneuver (ADIM), EFCD, and at rest multiple comparison mean difference for muscle thickness. Abbreviations: ADIM, abdominal drawing-in maneuver; EFCD, expiratory flow control device; EO-Th, External oblique thickness; IO-Th internal oblique thickness; TrA-Th, transversus abdominis thickness.

Sociodemographic data showed that the mean age of the participants was 29 ± 6 years, weight was 59 ± 9 kg, height was 1.65 ± 0.07 m, and body mass index (BMI) was 21.78 ± 2.54 kg/m^2^. Statistically significant differences were observed between ADIM, EFCD, and at rest condition for the thickness of the TrA (*p* = 0.001) and internal oblique (IO) (*p* = 0.039) (Table 1). Moreover, statistically significant differences for TrA at rest compared with the ADIM (*p*= 0.001), Cohen’s *d* = 2.183) and at rest and with the EFCD (*p* = 0.001), Cohen’s *d* = 2.843). In addition, between ADIM and EFCD were not statistically significant, although a moderate effect size was found (*p* = 0.055, Cohen’s *d* = 0.694). For the IO muscle thickness, significant differences was reported between the EFCD and at rest (*p* = 0.038, Cohen’s *d* = 0.081). The rest of the variables did not show any significant differences (Table 2, Figure 3).

## 4. Discussion

To the author’s knowledge, there was not prior evidence of the existence of research that evaluated the thickness of the lateral abdominal muscles during the use of the EFCD. Several authors employed the ultrasound imaging to evaluate the lateral abdominal musculature during an ADIM [22,26,29,30]. In addition, previous studies compared lateral abdominal musculature activation during an ADIM with activation during maximal expiration [9,15,21].

Whittaker et al.’s guidelines were employed to carry out the ultrasound imaging assessments of the lateral abdominal musculature activation. In the study by Hides et al., the use of ultrasound was validated to evaluate the contraction of the TrA and IO [30]. Moreover, the muscle thickness measurements for the TrA and IO obtained in this study are similar to those obtained during rest and contraction in other studies [29,30].

Ishida et al. [21] made a comparison between the changes in lateral abdominal musculature contraction during an ADIM with the changes during a maximal expiration, finding a greater contraction of all muscles during expiration. According to our findings, only in the case of the TrA was a tendency shown that supported a larger contraction during expiration with the EFCD than during the ADIM. The differences could be related to the fact that expiration through the EFCD does not require maximal expiration. Future studies could investigate the relationship between the intensity of the expiration and the response of the abdominal musculature. The percent of change in TrA and IO found by Ishida et al. [21] during the ADIM (56% and 14%, respectively) and during maximal expiration (89% and 29%) were similar to those found in this study, with a lower percent change in the IO during expiration with the EFCD. Our findings showed that there were not significant differences in the EO when comparing the increase in thickness at rest with that during the ADIM or during expiration with the EFCD. Ishida et al. [21] found an increase in EO thickness, as assessed by ultrasound imaging. However, the measurement of EO thickness by ultrasound has not showed a consistent relationship with its level of activation [19].

In the study by Miaki et al. [15], TrA activation during the ADIM is compared with expiratory training, finding greater activation with such training. Nevertheless, the differences in muscle thickness between that study and the present may be due to differences in the age of the study population: 29 ± 6 years in our study and 85 ± 6 years in the Miaki et al. study.

Regarding waist circumference, we did not find statistically significant differences in the ADIM group with respect to EFCD group. Choi et al. [27] reported that there is a relationship between abdominal muscle training and decreased waist circumference; however, it would be necessary to study in more depth the relationship between the type of contraction or exercise and the decrease in waist circumference. In addition, Rostami et al. [28] reported that waist circumference might be assumed as a good parameter to evaluate the abdominal muscles.

### Clinical Relevance

Our findings suggest that the EFCD may have benefits to the assessment and management in patients with disturbances related to abdominal wall muscles (e.g., pelvic floor disorders, lumbar pathology, respiratory disturbances, or sports populations). In addition, this EFCD could be useful in an isolated manner, or in addition to traditional approaches, such as manual therapy or sport performance, prevention, and rehabilitation.

As limitations of this study, we should mention the difficulty to standardize the drawing-in maneuver, which is dependent on the ability of the subjects, as well as the impossibility to standardize the execution intensity of the ADIM and expiration through the EFCD. For this study, additional information of the subjects’ lifestyle was not recorded, such as sports activities or hours spent sitting per day. Further studies need to be developed in order to evaluate the effectiveness about this method for the abdominal muscles in the sports field. In addition, it would be interesting to evaluate the effect of the drawing-in maneuver and EFCD on these structures, as well as the consequences of training in a given period of time.

## 5. Conclusions

We found statistically significant differences in the increase of the thickness of the TrA and IO muscles during the use of the EFCD and the ADIM with respect to rest. In addition, for the TrA, statistically significant differences were found during expiration with the EFCD with respect to the ADIM. Therefore, expiration with the EFCD can be a useful method for TrA activation.

## Figures and Tables

**Table 1:** One-way ANOVA inter-group comparation and percent of change. Muscle thickness and waist circumference.

Variable	Mean ± SD	% of Change	*F*	*p* Value
TrA thickness (mm)			40.103	0.001
Rest	0.33 ± 0.08	*n*/*a*		
ADIM	0.54 ± 0.11	66.65 ± 28.93		
EFCD	0.62 ± 0.12	92.95 ± 37.95		
IO thickness (mm)			3.416	0.039
Rest	0.66 ± 0.12	*n*/*a*		
ADIM	0.74 ± 0.13	13.78 ± 18.02		
EFCD	0.79 ± 0.19	19.28 ± 22.21		
EO thickness (mm)			0.809	0.450
Rest	0.52 ± 0.08	*n*/*a*		
ADIM	0.51 ± 0.10	−0.47 ± 14.37		
EFCD	0.48 ± 0.10	−6.24 ± 14.87		
Waist circumference (cm)			0.519	0.598
Rest	74.99 ± 8.01	*n*/*a*		
ADIM	72.49 ± 7.97	−3.36 ± 1.37		
EFCD	73.32 ± 8.35	−2.27 ± 2.39		

Values are mean ± SD unless otherwise indicated. Abbreviature: ADIM, abdominal drawing-in maneuver; EFCD, expiratory flow control device; EO, external oblique; IO, internal oblique, SD, standard deviation; TrA, transversus abdominis.

**Table 2:** Abdominal drawing-in maneuver (ADIM), EFCD, and at rest multiple comparison *p* values and effect sizes for muscle thickness and waist circumference.

Variable	Intervention	Intervention	*p* Value (Cohen-s d)
TrA thickness (mm)			
	Rest	ADIM	0.001 (2.183)
	Rest	EFCD	0.001 (2.843)
	ADIM	EFCD	0.055 (0.694)
IO thickness (mm)			
	Rest	ADIM	0.277 (0.639)
	Rest	EFCD	0.038 (0.081)
	ADIM	EFCD	1.000 (0.307)
EO thickness (mm)			
	Rest	ADIM	1.000 (0.110)
	Rest	EFCD	0.754 (0.441)
	ADIM	EFCD	0.917 (0.300)
Waist circumference			
	Rest	ADIM	0.964 (0.312)
	Rest	EFCD	1.000 (0.204)
	ADIM	EFCD	1.000 (0.101)

Values are mean ± SD unless otherwise indicated. Abbreviatures: ADIM, abdominal drawing-in maneuver; EFCD, expiratory flow control device; EO, external oblique; IO, internal oblique, SD, standard deviation; TrA, transversus abdominis.

## References

[1] Paulsen F., Waschke J. (2013). English: General Anatomy and Musculoskeletal System. Sobotta Atlas of Human Anatomy.

[2] Shih-Lin H., Oda H., Shirahata S., Watanabe M., Sasaki M. (2018). Effects of neuromuscular training on core stability. J. Phys. Ther. Sci..

[3] Hides J., Wilson S., Stanton W., McMahon S., Keto H., McMahon K., Bryant M., Richardson C. (2006). An MRI investigation into the function of the transversus abdominis muscle during “drawing-in” of the abdominal wall. Spine (Phila Pa 1976).

[4] Urquhart D.M., Hodges P.W., Allen T.J., Story I.H. (2005). Abdominal muscle recruitment during a range of voluntary exercises. Man Ther..

[5] Arjmand N., Shirazi-Adl A. (2006). Role of intra-abdominal pressure in the unloading and stabilization of the human spine during static lifting tasks. Eur. Spine J..

[6] Yoshimura N., Tomita T., Abe T., Easton P.A., Kusuhara N. (2017). Differential respiratory activity of four abdominal muscles in humans. J. Appl. Physiol..

[7] Sapsford R.R., Hodges P.W., Richardson C.A., Cooper D.H., Markwell S.J., Jull G.A. (2001). Co-activation of the abdominal and pelvic floor muscles during voluntary exercises. Neurourol. Urodyn..

[8] Junginger B., Baessler K., Sapsford R., Hodges P.W. (2010). Effect of abdominal and pelvic floor tasks on muscle activity, abdominal pressure and bladder neck. Int. Urogynecol. J..

[9] Ishida H., Watanabe S. (2013). Changes in lateral abdominal muscles’ thickness immediately after the abdominal drawing-in maneuver and maximum expiration. J. Bodyw. Mov. Ther..

[10] Kim D.-H., Kim T.-H. (2019). Effects of abdominal drawing-in maneuver with pressure biofeedback, foam-roller and quadruped on lumbopelvic stability and muscle activities in lumbar rotation syndrome. J. Exerc. Rehabil..

[11] Lee J.-Y., Lee D.-Y. (2018). The effect of therapeutic abdominal drawing-in maneuver using ultrasonography on lateral abdominal muscle thickness and balance. J. Back Musculoskelet Rehabil..

[12] Cho M. (2015). The effects of bridge exercise with the abdominal drawing-in maneuver on an unstable surface on the abdominal muscle thickness of healthy adults. J. Phys. Ther. Sci..

[13] Kim C.-Y., Lee J.-S., Kim H.-D., Kim I.-S. (2015). Effects of the combination of respiratory muscle training and abdominal drawing-in maneuver on respiratory muscle activity in patients with post-stroke hemiplegia: A pilot randomized controlled trial. Top Stroke Rehabil..

[14] Richardson C., Hodges P.W., Hides J. (2004). Manipulation Association of Chartered Physiotherapists. Therapeutic Exercise for Lumbopelvic Stabilization: A Motor Control Approach for the Treatment and Prevention of Low Back Pain.

[15] Miaki H., Madokoro S., Yokogawa M., Sugimoto T., Nakagawa T. (2018). Changes in thickness of the transversus abdominis during the abdominal drawing-in manoeuvre and expiratory muscle training in elderly people. J. Phys. Ther. Sci..

[16] Cheminal R., Hotton C., Delorme E., Trackoen G., Pasquale J., Mege J.-L. (2008). Description et résultat d’une étude prospective portant sur une nouvelle méthode de kinésithérapie dans la prise en charge de l’incontinence urinaire postprostatectomie Description and results of a prospective study on a new physiotherapy method in the ma. Progrès en Urol..

[17] Guillarme L., Delorme E. (2004). Rééducation Thoraco-Abdomino-Pelvienne par le Concept ABDO-MG: La Renaissance Abdominale par le Souffle [Thoraco-Abdomino-Pelvic Reeducation by ABDO-MG Concept: Abdominal Rebirth by Blowing].

[18] Taghipour M., Mohseni Bandpei M.A., Behtash H., Abdollahi I., Rajabzadeh F., Pourahmadi M.R., Emami M. (2019). Reliability of real-time ultrasound imaging for the assessment of trunk stabilizer muscles: A systematic review of the literature. J. Ultrasound Med..

[19] Hodges P.W., Pengel L.H.M., Herbert R.D., Gandevia S.C. (2003). Measurement of muscle contraction with ultrasound imaging. Muscle Nerve..

[20] Park S. (2013). Reliability of Ultrasound Imaging of the Transversus Deep Abdominial, Internal Oblique and External Oblique Muscles of Patients with Low Back Pain Performing the Drawing-in Maneuver. J. Phys. Ther. Sci..

[21] Ishida H., Hirose R., Watanabe S. (2012). Comparison of changes in the contraction of the lateral abdominal muscles between the abdominal drawing-in maneuver and breathe held at the maximum expiratory level. Man Ther..

[22] Tahan N., Rasouli O., Arab A.M., Khademi K., Samani E.N. (2014). Reliability of the ultrasound measurements of abdominal muscles activity when activated with and without pelvic floor muscles contraction. J. Back Musculoskelet Rehabil..

[23] Beazell J.R., Grindstaff T.L., Hart J.M., Magrum E.M., Cullaty M., Shen F.H. (2011). Changes in lateral abdominal muscle thickness during an abdominal drawing-in maneuver in individuals with and without low back pain. Res. Sport Med..

[24] Whittaker J.L., Warner M.B., Stokes M. (2013). Comparison of the Sonographic Features of the Abdominal Wall Muscles and Connective Tissues in Individuals with and without Lumbopelvic Pain. J. Orthop. Sport Phys. Ther..

[25] Talasz H., Kremser C., Kofler M., Kalchschmid E., Lechleitner M., Rudisch A. (2011). Phase-locked parallel movement of diaphragm and pelvic floor during breathing and coughing-a dynamic MRI investigation in healthy females. Int. Urogynecol. J..

[26] Miltenberger C.E., Deiters H.M., Toro YMDel Pulliam J.N. (2005). The Use of Ultrasound Imaging of the Abdominal Drawing-in Maneuver in Subjects with Low Back Pain. J. Orthop. Sport Phys. Ther..

[27] Choi E.J., Kim Y.J., Lee S.Y. (2018). Effects of electrical muscle stimulation on waist circumference in adults with abdominal obesity: A randomized, double-blind, sham-controlled trial. J. Nepal. Med. Assoc..

[28] Rostami M., Yekta AH A., Noormohammadpour P., Farahbakhsh F., Kordi M., Kordi R. (2013). Relations between Lateral Abdominal Muscles Thickness, Body Mass Index, Waist Circumference and Skin Fold Thickness. Acta Med. Iran..

[29] Jhu J.-L., Chai H.-M., Jan M.-H., Wang C.-L., Shau Y.-W., Wang S.-F. (2012). Reliability and Relationship between 2 Measurements of Transversus Abdominis Dimension Taken during an Abdominal Drawing-in Maneuver Using a Novel Approach of Ultrasound Imaging. J. Orthop. Sport Phys. Ther..

[30] Hides J.A., Miokovic T., Belavý D.L., Stanton W.R., Richardson C.A. (2007). Ultrasound Imaging Assessment of Abdominal Muscle Function during Drawing-in of the Abdominal Wall: An Intrarater Reliability Study. J. Orthop. Sport Phys. Ther..

